# Bilobar hepatic hydatid cyst: a rare encounter with a silent invader

**DOI:** 10.1093/jscr/rjaf262

**Published:** 2025-04-30

**Authors:** Suman Kumar Khadka, Aashish Baniya, Suresh Prasad Shah, Dinesh Nalbo, Bikram Bhandari

**Affiliations:** Department of Surgery, B.P. Koirala Institute of Health Sciences, Ghopa Camp, Dharan 18, Sunsari District, 56700, Nepal; B.P. Koirala Institute of Health Sciences, Ghopa Camp, Dharan 18, Sunsari District, 56700, Nepal; Department of Surgery, B.P. Koirala Institute of Health Sciences, Ghopa Camp, Dharan 18, Sunsari District, 56700, Nepal; Department of Surgery, Birat Medical College Teaching Hospital, Tankisinuwari 2, Budhiganga, Morang District, 56613, Nepal; B.P. Koirala Institute of Health Sciences, Ghopa Camp, Dharan 18, Sunsari District, 56700, Nepal

**Keywords:** hydatid disease, *Echinococcus*, bilobar involvement, laparoscopic pericystectomy

## Abstract

Hydatid disease, caused by *Echinococcus* species, primarily affects the liver and lungs. It is endemic in many regions, including Nepal, and often remains asymptomatic for years. This report presents a rare case of hepatic hydatid cysts affecting both lobes. A 47-year-old male with a history of alcohol consumption presented with right upper abdominal pain and vomiting. Imaging revealed two thick-walled, multiloculated cystic lesions in both hepatic lobes, classified as Stage CE 2 (WHO-IWGE). The patient was started on albendazole and underwent a successful laparoscopic pericystectomy. While most hepatic hydatid cases involve a single cyst in the right lobe, bilobar involvement is uncommon. Imaging plays a crucial role in diagnosis, and surgical intervention remains the gold standard for treatment. Early recognition and management are vital for preventing complications. This case highlights the need for vigilance in endemic regions to ensure timely diagnosis and optimal outcomes.

## Introduction

Hydatid disease, also known as echinococcosis or hydatidosis, is caused by an infection with the larval (metacestode) stage of the tapeworm *Echinococcus* [1]. The two main species responsible for hydatid disease in humans are *Echinococcus granulosus* and *Echinococcus multilocularis*, with the infection progressing slowly over time [1]. Humans and sheep serve as intermediate hosts in the parasite’s lifecycle [2]. In humans, the liver is the most commonly affected organ, accounting for 50%–75% of cases, followed by the lungs (25%) and the arterial system (5%–10%) [2]. While hepatic involvement predominates in adults, pulmonary involvement is more frequent in children [3].

Hydatid disease is endemic in several regions, including the Mediterranean, the Middle East, the Far East, and South Africa [4]. It often remains asymptomatic for an extended period due to the slow growth of cysts. Common symptoms include fatigue and abdominal pain, while complications such as jaundice, hepatomegaly, or anaphylaxis may arise from cyst leakage or rupture [5].

This case report describes an unusual presentation of a hydatid cyst affecting both lobes of the liver in a 47-year-old male patient.

## Case presentation

A 47-year-old male with a history of chronic alcohol consumption and smoking presented to the outpatient department of our institution, Dharan, Nepal. His chief complaint was acute-onset, dull aching pain in the right upper abdomen persisting for 15 days. The pain was nonradiating and had no associated symptoms. He also reported three episodes of vomiting over the past two days. The vomitus was nonbilious, nonprojectile, foul-smelling, contained food particles, and was not mixed with blood.

His past medical and family history were unremarkable. On examination, the patient was conscious, alert, and well-oriented to time, place, and person, with a Glasgow Coma Scale score of 15/15. His vital signs were within normal limits. A general physical examination revealed no pallor, icterus, clubbing, lymphadenopathy, cyanosis, edema, or dehydration. Systemic examinations of the respiratory, cardiovascular, and central nervous systems were unremarkable. However, an abdominal examination revealed tenderness in the right subcostal region.

Given the clinical presentation, a hepatobiliary pathology was suspected. The patient was advised to undergo radiological investigations, including an ultrasound of the abdomen and pelvis, along with routine hematological tests, liver and renal function tests, electrocardiography, and echocardiography to rule out any concurrent infective or inflammatory lesions.

All routine laboratory investigations were within normal limits. The echocardiogram revealed mild mitral regurgitation and moderate tricuspid regurgitation. Ultrasonography (USG) of the abdomen and pelvis identified two well-defined, thick-walled cystic lesions in both lobes of the liver with multiple internal septations, the largest being in the left lobe, with no internal vascularity—findings suggestive of hepatic hydatid cysts.

As per the World Health Organization Informal Working Group on Echinococcosis (WHO-IWGE) classification of cystic echinococcosis, the lesion was classified as Stage CE 2 (active stage-honeycomb appearance with internal septations).

Following USG, a contrast-enhanced computed tomography (CECT) scan of the abdomen and pelvis was performed for further evaluation of the cystic lesions, including their size, location, wall thickness, and septations. The CECT scan revealed:

- A well-defined, thick-walled (maximum thickness ~ 4.5 mm), multiloculated cystic lesion measuring ~8.9 × 9.7 × 9.4 cm (AP × TR × CC) with multiple thick enhancing septa (giving a honeycomb appearance) involving segments II and III of the left lobe. Laterally, it was abutting the left portal vein and inferiorly abutting the main portal vein.
- Another well-defined, thick-walled (maximum thickness ~6.5 mm) cystic lesion measuring ~5.6 × 4.4 × 4 cm (AP × TR × CC) with enhancing septa located in segment V of the right lobe.

Based on the clinical presentation and imaging findings, the patient was diagnosed with Stage CE 2 WHO-IWGE classification hepatic hydatid cyst with bilobar involvement.

The patient was initially started on oral albendazole 400 mg twice daily as antihelminthic therapy. Simultaneously, he was planned for laparoscopic pericystectomy. The intraoperative and postoperative periods were uneventful, with complete cyst excision achieved. Postoperatively, the patient received a full course of antibiotic therapy along with analgesics as needed.

## Discussion

Echinococcosis is a zoonotic disease transmitted primarily by dogs, with humans and sheep serving as accidental hosts [6]. It is endemic in various parts of the world, particularly in Asia, with the liver being the most commonly affected organ, followed by the lungs [6]. Most patients (72%) present with a single cyst in the right hepatic lobe, and 87% exhibit single-organ involvement [6]. However, our case is unusual, as the cysts were present in both hepatic lobes.

The clinical presentation of hydatid disease depends on several factors, including the organ involved, cyst size and location, interactions with adjacent structures, and potential complications [3]. In our patient, symptoms were limited to abdominal pain and a few episodes of vomiting, with no significant complications. As hepatic involvement is most common in adults, hepatic dysfunction-related symptoms predominate in such cases.

**Figure 1:**
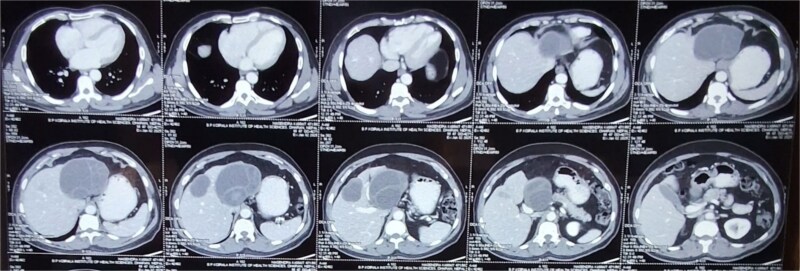
CECT abdomen pelvis showing bilobar hepatic hydatid cyst.

For diagnosing hepatic hydatid disease, imaging studies play a crucial role. Computed tomography (CT) is the investigation of choice (as shown in Fig. 1), with a specificity of 98%, while USG has a sensitivity of 90%–95% [1]. Both modalities are highly accurate in diagnosing hepatic hydatidosis, allowing for the assessment of cyst structure, number, location, and potential complications.

**Figure 2:**
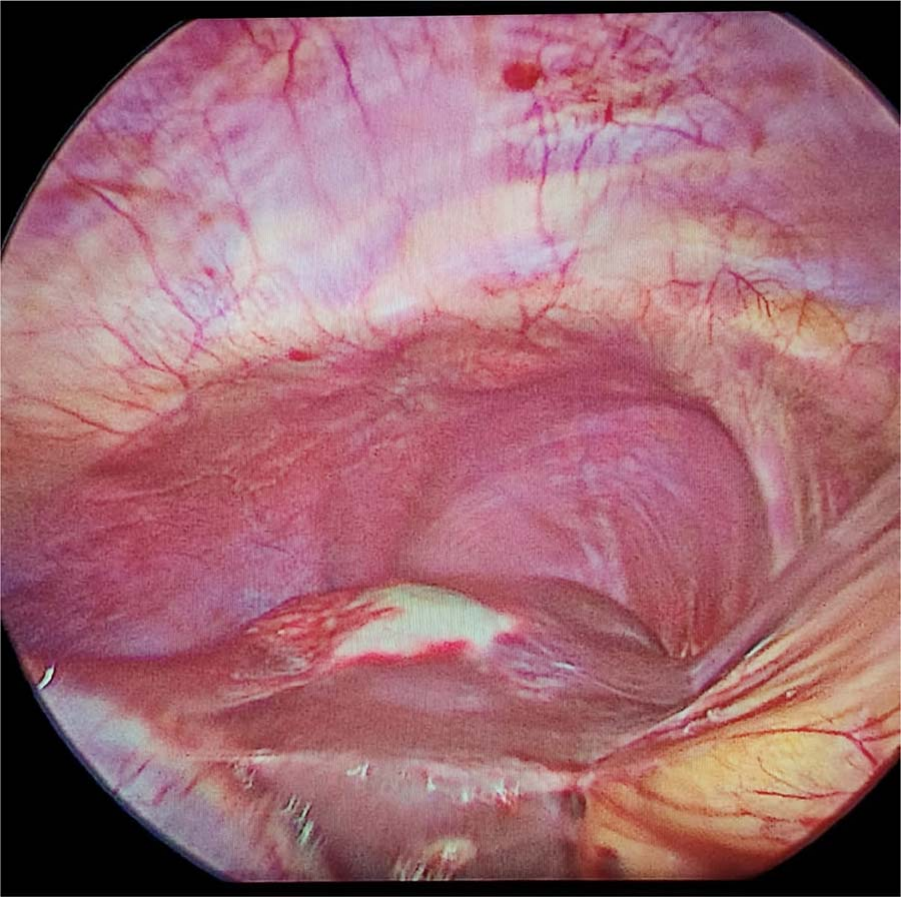
Laparoscopic view of hepatic hydatid lesion (white focus on the liver surface).

**Figure 3:**
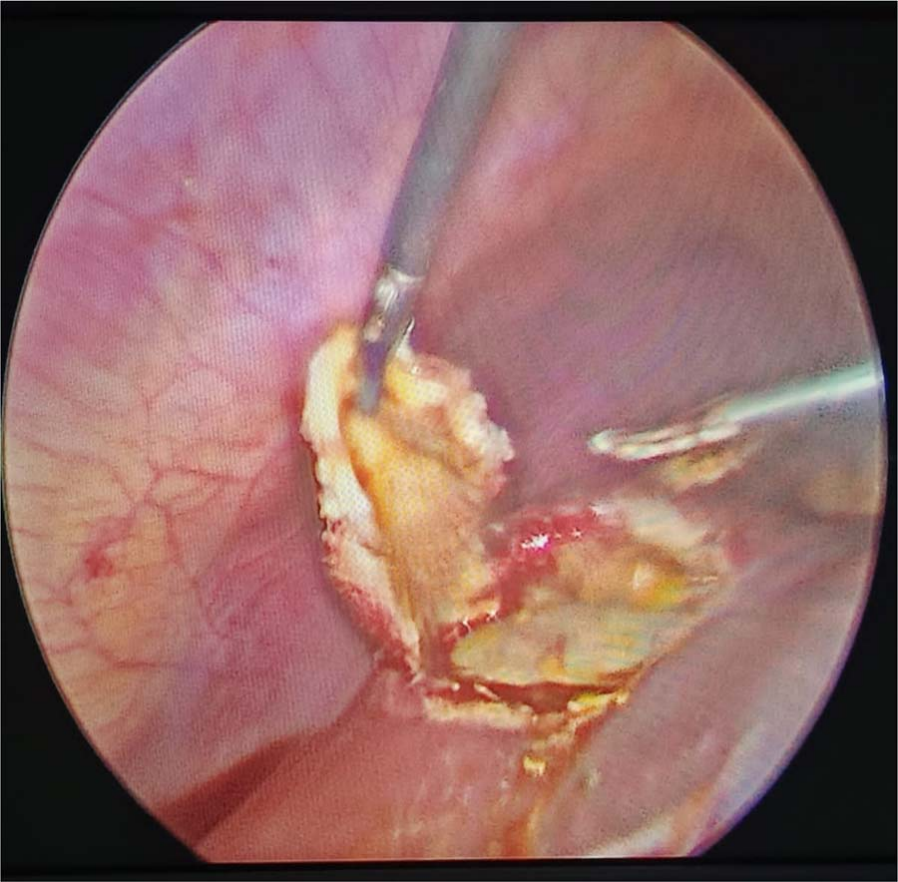
Laparoscopic pericystectomy for hepatic hydatid cyst.

**Figure 4:**
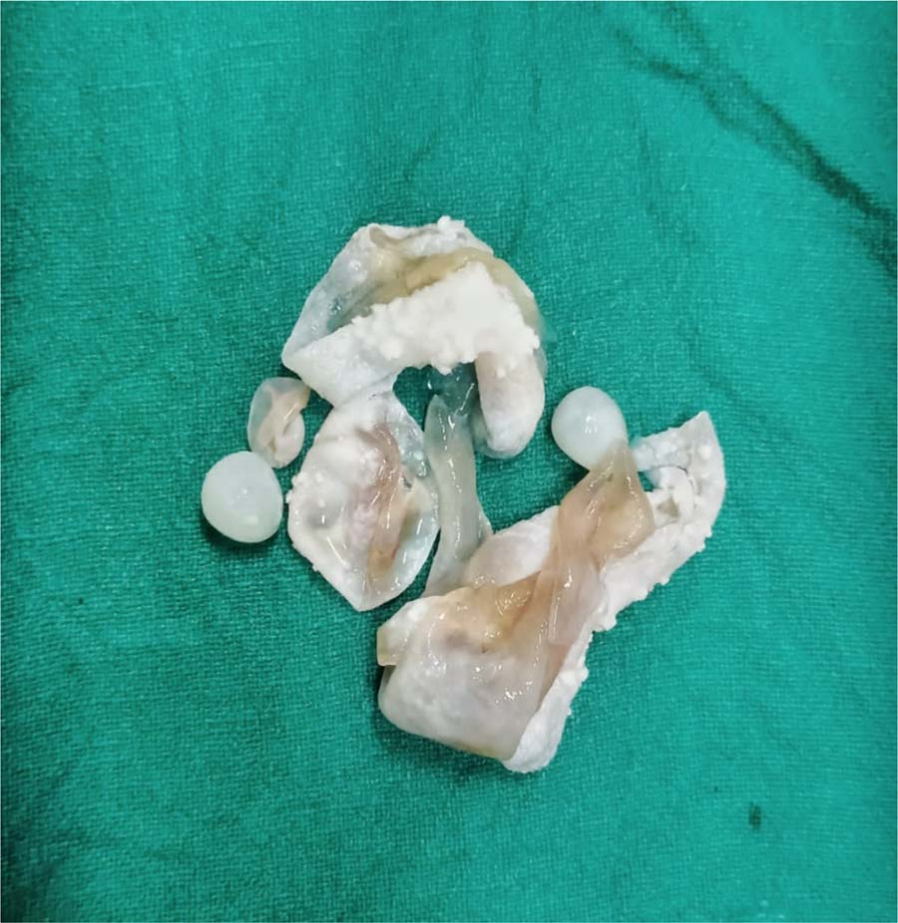
Excised hydatid cysts.

The treatment of cystic hydatid disease includes percutaneous drainage, medical therapy, and surgical intervention. While percutaneous aspiration-injection-reaspiration and albendazole therapy are viable options, surgery remains the preferred treatment for complicated cysts, offering the potential for complete cyst removal and full recovery [3, 6]. In our case, laparoscopic pericystectomy was successfully performed, leading to a favorable postoperative outcome (Figs 2–4).

## Conclusion

This case underscores the importance of early recognition and appropriate management of hepatic hydatid disease. While most cases present with a single cyst in the right lobe, the presence of cysts in both lobes, as observed in our patient, is relatively rare. Imaging modalities such as USG and CT play a crucial role in confirming the diagnosis and guiding surgical intervention. Timely surgical management, combined with adjunctive antihelminthic therapy, remains the cornerstone of treatment, ensuring complete cyst eradication and reducing the risk of recurrence. Given the endemic nature of echinococcosis, clinicians should maintain a high index of suspicion in patients presenting with unexplained abdominal symptoms, enabling prompt diagnosis and optimal patient outcomes.
